# Diagnostic Value of Combinatorial Markers in Colorectal Carcinoma

**DOI:** 10.3389/fonc.2020.00832

**Published:** 2020-05-22

**Authors:** Veronika Voronova, Peter Glybochko, Andrey Svistunov, Viktor Fomin, Philipp Kopylov, Peter Tzarkov, Alexey Egorov, Evgenij Gitel, Aligeydar Ragimov, Alexander Boroda, Elena Poddubskaya, Marina Sekacheva

**Affiliations:** ^1^M&S Decisions LLC, Moscow, Russia; ^2^I.M. Sechenov First Moscow State Medical University, Moscow, Russia

**Keywords:** diagnostics, biomarkers, colorectal cancer, machine learning, carcinoembryonic antigen, apolipoproteins

## Abstract

**Objectives:** Blood-based tests have been shown to be an effective strategy for colorectal cancer (CRC) detection in screening programs. This study was aimed to test the performance of 20 blood markers including tumor antigens, inflammatory markers, and apolipoproteins as well as their combinations.

**Methods:** In total 203 healthy volunteers and 102 patients with CRC were enrolled into the study. Differences between healthy and cancer subjects were evaluated using Wilcoxon rank-sum test. Several multivariate classification algorithms were employed using information about different combinations of biomarkers altered in CRC patients as well as age and gender of the subjects; random sub-sampling cross-validation was done to overcome overfitting problem. Diagnostic performance of single biomarkers and multivariate classification models was evaluated by receiver operating characteristic (ROC) analysis.

**Results:** Of 20 biomarkers, 16 were significantly different between the groups (*p*-value ≤ 0.001); ApoA1, ApoA2 and ApoA4 levels were decreased, whereas levels of tumor antigens (*e.g*. carcinoembriogenic antigen) and inflammatory markers (e.g., C-reactive protein) were increased in CRC patients vs. healthy subjects. Combinatorial markers including information about all 16 significant analytes, age and gender of patients, demonstrated better performance over single biomarkers with average accuracy on test datasets ≥95% and area under ROC curve (AUROC) ≥98%.

**Conclusions:** Combinatorial approach was shown to be a valid strategy to improve performance of blood-based CRC diagnostics. Further evaluation of the proposed models in screening programs will be performed to gain a better understanding of their diagnostic value.

## Introduction

Colorectal cancer (CRC) is the third most commonly diagnosed malignancy worldwide with the highest prevalence in developed countries (1). In 2018, the predicted total mortality rates in the Russian Federation were 158.5/100,000 men and 84.1/100,000 women (2). Early diagnosis of cancer represents an effective way to reduce mortality rates, however, since clinical symptoms are often minor and non-specific until advanced disease stages, dedicated screening programs are required (3).

Several instrumental methods are currently used to diagnose CRC, including colonoscopy, computer tomography (CT), colonoscopy, flexible sigmoidoscopy etc. (4, 5). While these methods are required to confirm diagnosis, their usage in screening programs is limited due to invasiveness, labor intensiveness, risk of complications and the need for specific equipment. Additionally, several non-invasive methods such as fecal immunochemical test (FIT), fecal occult blood testing (FOBT) can be used (4, 6), however, high false positive rates are an important disadvantage of these tests (7, 8). DNA-based methods represent another strategy of CRC detection, but despite the diagnostic advantage over FOBT these systems cannot be used in screening programs due to their expense (9).

Blood-based tests would be the most suitable option for massive screening programs, since they can be easily combined with other biochemical assays. Several blood-based biomarkers, including carcioembriogenic antigen (CEA) and carbohydrate antigen (CA) 19-9 are well established in clinical practice, howbeit, low specificity and sensitivity are key limitations of these tests (10). Recent advances in -omics technologies enabled discovery of new potential biomarkers, including different proteins (11), circulating tumor DNA (12, 13) or microRNA (14) and circulating tumor cells (15) as well as numerous metabolites (16, 17) and transcriptional biomarkers (18). Despite many of these biomarkers demonstrated high diagnostic potential in retrospective proof-of-concept studies, further research is required to determine their clinical validity and utility (11). Another challenges, limiting extensive use of these biomarkers in routine practice nowadays, are their expensiveness and lack of reproducibility (11).

An alternative strategy of the screening optimization is exploiting multifactorial approaches, implying development of multivariate classification models, which can be used to calculate probability of having the disease based on measurements of several biomarkers (10, 19). Such biomarkers may demonstrate higher diagnostic performance compared to single analytes due to more comprehensive reflection of complex and diverse mechanisms of carcinogenesis and multiple metabolic, genetic and structural alternations in cancer cells (10). The current work is aimed to assess the diagnostic potential of multiple biomarkers, including oncofetal proteins, inflammation, and vascularization markers, adhesion molecules and their combination to evaluate the CRC risk.

## Materials and Methods

### Patients, Sampling and Measurements

The study was approved by the Local Ethics Committee of I.M. Sechenov First Moscow State Medical University. All patients were given an informed consent to participate in the study. In total 102 patients with histologically-confirmed CRC (16 patients with T1-2, 86 patients with T3-4) and 203 healthy subjects were included in the analysis. Serum samples were collected at Sechenov University Hospital after overnight fasting and sent to the Hospital laboratory. Samples were stored at −70°C in liquid nitrogen until analyzed.

In total 20 biomarkers were measured including apolipoproteins A1, A2, B (ApoA1, ApoA2, ApoB), alpha-fetoprotein (AFP), beta 2 microglobulin (B2M), carbohydrate antigen 19-9 (CA 19-9), cancer antigens 15-3 and 125 (CA 15-3, CA 125), carcinoembryonic antigen (CEA), cytokeratin 19-fragments (CYFRA 21-1), human epididymis protein 4 (HE4), human-specific C-reactive protein (hsCRP), D-dimer, leucine-rich alpha-2-glycoprotein 1 (LRG 1), total prostate-specific antigen (PSA), regulated on activation, normal T cell expressed and secreted (RANTES) soluble vascular cell adhesion molecule 1 (sVCAM 1), transthyretin (TTR), vascular endothelial growth factor receptor 1 (VEGFR 1). Biomarker levels were measured in all 305 samples, except total PSA, which was only analyzed in serum samples obtained from men.

Sandwich enzyme-linked immunosorbent assay (ELISA) was used to analyze RANTES, sVCAM-1, VEGFR-1, ApoA4, LRG-1 (Quantikine® kits, R&D systems, US) with Biochrom Anthos 2020 microplate reader (Biochrom, UK); AFP, CA15-3, CA19-9, CA125, HE4, CEA, CYFRA21-1, and total PSA were measured using Elecsys® sandwich electrochemiluminiscent assay (ECLIA) on the Cobas e411 analyzer (Roche diagnostics, Germany); hsCRP, ApoA1, ApoB, TTR were measured on Advia 1800 auto-analyzer by immunoturbodimeric method (Siemens Healthcare, Germany); B2M and Ddimer were measured by sandwich chemiluminescent assay (CLIA) on Immulite 2000 auto-analyzer (Siemens Medical Solutions, USA); ApoA2 was measured using enzymatic colorimetric method (Randox laboratories, UK).

### Statistical Methods

All data processing, statistical and visualization procedures were performed using R statistical software (v.3.5.1) (20). R-based packages randomForest (v.4.6-14), MASS (v.7.3-50), e1071 (v.1.7-2), stats (v.3.5.1) and caret (v.6.0-84) were used for development of combinatorial biomarkers; sensitivity analysis of the developed biomarkers was done using R-based mmpf package (v.0.0.5); R-based pROC package was used to perform ROC analysis (v.1.15.3).

Biomarker values were log-transformed prior to analysis. At first, the significance of single biomarkers was evaluated using Mann-Whitney *U*-test and the diagnostic value of each biomarker was assessed via receiver operating characteristics (ROC) analysis; sensitivity, specificity, and diagnostic accuracy at optimal cut-off values as well as area under ROC curve (AUROC) were calculated. Influence of subject characteristics (gender and age) on biomarker levels in healthy and CRC groups was evaluated via analysis of covariance (ANCOVA) using generalized linear models.

Secondly, classification models were assembled based on the measurements of biomarkers, which were significantly different between healthy subjects and CRC patients (*p*-value<0.05) and demonstrated discriminative ability (AUROC > 0.6). Patient characteristics (age and gender) were also tested as predictors. Several classification algorithms including random forest (RF), support vector machine (SVM), linear discriminant analysis (LDA), and naïve Bayes classifier (NBC), as well as multiple logistic regression (MLR) (21) were trained using the whole dataset and their discriminative ability was assessed via ROC analysis, similar to single biomarkers. Accuracy of model-predicted probabilities of having the disease was evaluated using Brier score. To detect overfitting of classification models a 100-times repeated random 5-fold sub-sampling cross-validation was performed. Sensitivity of the model predictions to changes in values of single biomarkers and patient characteristics was evaluated using model-agnostic permutation importance method (22).

Finally, all possible classification models, exploiting information about one to five biomarkers and patient characteristics, were trained and their diagnostic performance was assessed.

## Results

### Diagnostic Accuracy of Single Biomarkers

**Figure 1:**
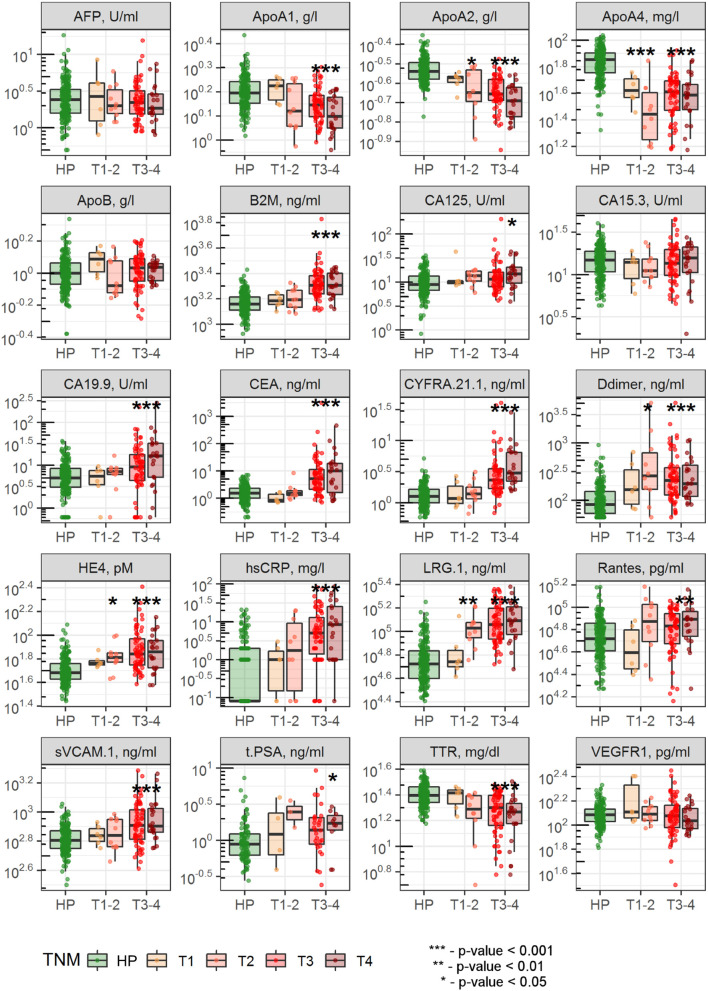
Comparison of biomarker levels between healthy subjects and patients with early and advanced CRC stages. Dots indicate individual patient data; differences between healthy subjects and CRC patients with stages T1-T2 or T3-T4 were evaluated using Wilcoxon test with Bonferroni correction for multiple testing.

Comparison of the biomarker levels in healthy subjects and CRC patients is presented in Figure 1 and Table 1. Among considered analytes AFP, ApoB, CA 15-3, and VEGFR 1 were not significantly different between the two groups; ApoA1 and ApoA2 levels were lower in CRC group compared to healthy subjects; levels of the rest biomarkers were higher in CRC vs. healthy group (Table 1). While disease stratification to early (T1-T2) and advanced (T3-T4) stages, levels of ApoA2, ApoA4, Ddimer, HE4, and LRG 1 were found to be significantly changed in both early and advanced CRC stages (Figure 1). As can be seen from Table 1, mean age of CRC patients was higher compared to healthy subjects (48 ± 6.33 and 63 ± 12.4 years, respectively, *p*-value < 0.001); in accordance to ANCOVA results, significant differences in biomarker levels persisted after age and gender adjustment (Table S1).

**Table 1:** Diagnostic performance of biomarkers for CRC diagnosis, ranged by AUROC.

**Characteristic/Biomarker**	**Units**	**Healthy subjects, mean ± SD (*n* = 203)**	**CRC patients, mean ± SD (*n* = 102)**	***p*-value^a^**	**AUROC**	**Specificity, %**	**Sensitivity, %**	**Accuracy, %**
Age	Years	48 ± 6.33	63 ± 12.4	<0.001	-	-	-	-
Gender (F/M)	-	104/99	56/46	0.63^b^	-	-	-	-
Stage	-	-	T1 (*n* = 6); T2 (*n* = 10); T3 (63); T4 (23)	-	-	-	-	-
ApoA4	mg/l	69.22 ± 16.88	39.68 ± 14.8	<0.001	0.9	74	93	81
LRG 1	ng/ml	58847.79 ± 25516.45	121289 ± 48054.03	<0.001	0.89	82	83	83
ApoA2	g/l	0.3 ± 0.04	0.23 ± 0.05	<0.001	0.87	94	65	84
B2M	ng/ml	1477.29 ± 293.83	2056.66 ± 675.6	<0.001	0.83	80	73	78
CYFRA 21-1	ng/ml	1.37 ± 0.57	4.04 ± 6.02	<0.001	0.82	79	72	77
Ddimer	ng/ml	119.75 ± 103.78	435.54 ± 750.57	<0.001	0.8	64	82	70
HE 4	pM	51.43 ± 14.24	76.75 ± 34.23	<0.001	0.79	67	80	72
hsCRP	mg/l	1.77 ± 3.51	11.27 ± 16.95	<0.001	0.79	85	62	77
TTR	mg/dl	25.64 ± 4.88	19.25 ± 6.54	<0.001	0.77	85	57	76
CEA	ng/ml	1.86 ± 1.22	47.39 ± 306.02	<0.001	0.75	92	55	79
sVCAM 1	ng/ml	658.37 ± 131.52	848.68 ± 293.71	<0.001	0.72	86	50	74
ApoA1	g/l	1.6 ± 0.24	1.4 ± 0.24	<0.001	0.71	67	68	67
PSA	ng/ml	1.13 ± 0.97	1.9 ± 1.61	0.003	0.7	80	63	74
CA 19-9	U/ml	6.6 ± 5.73	18.77 ± 37.04	<0.001	0.66	86	44	72
CA 125	U/ml	10.69 ± 6.03	16.95 ± 21.81	0.001	0.64	58	65	60
Rantes	pg/ml	57217.76 ± 23360.49	69108.26 ± 28509.26	0.003	0.64	68	60	65
AFP	U/ml	2.84 ± 1.96	2.73 ± 2.13	1	0.55	49	65	54
ApoB	g/l	1.03 ± 0.25	1.06 ± 0.24	1	0.55	64	50	59
CA 15-3	U/ml	15.13 ± 6.33	15.27 ± 8.08	1	0.52	76	32	61
VEGFR 1	pg/ml	122.76 ± 24.08	126.42 ± 43.4	1	0.52	87	26	67

a calculated by Wilcoxon test with Bonferroni correction for multiple comparison;

b *calculated by two-proportions Z-test*.

**Figure 2:**
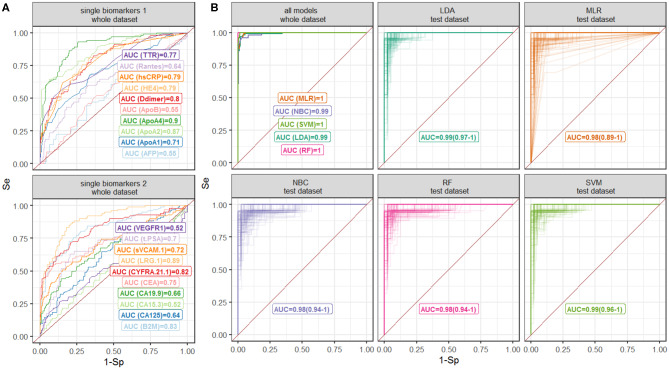
ROC curves for the **(A)** single-biomarker based tests and **(B)** multivariate classification models. Different models are shown by color. Numbers denote AUROC values; 90% confidence intervals for validation are shown in brackets.

Diagnostic accuracy of single biomarkers was assessed using the data, collected from all CRC patients simultaneously (Table 1) as well as separately from patients with early and advanced CRC stages (Tables S2, S3, Figure S1). The highest diagnostic performance was demonstrated for ApoA4, LRG 1, and ApoA2 with AUROC 0.9, 0.89, and 0.87, respectively (Table 1, Figure 2), which can be explained by their good performance in patients with both early and advanced stages; as expected, CRC-specific biomarkers, such as CEA and CA 19-9 demonstrated good performance only in CRC patients with advanced stages.

Diagnostic performance of AFP, ApoB, CA 15-3, and VEGFR 1 was poor (AUROC = 0.55, 0.55, 0.52, and 0.52, respectively, Table 1) and, hence, these biomarkers were excluded from further analysis. Total PSA measurements were not used for classification models, since the information was not available for all patients.

### Diagnostic Accuracy of Multivariate Classification Models

Measurements of 15 biomarkers, selected on the last step, were used to train classification models. Diagnostic performance of classification models as well as results of cross-validation are reported in Table 2; ROC curves are summarized in Figure 2. All multivariate classification models demonstrated better performance compared to single-marker-based tests while a whole dataset was used (AUROC ≥ 0.99, specificity and sensitivity ≥95%). In cross-validation exercise, MLR, NBC, and RF demonstrated higher variability in diagnostic performance compared to SVM and LDA.

**Table 2:** Diagnostic performance of 15-biomarker models for CRC diagnosis.

	**Full dataset**				**Cross-validation (test dataset)**			
	**AUROC**	**Specificity, %**	**Sensitivity, %**	**Accuracy, %**	**AUROC**	**Specificity, % (90% CI)**	**Sensitivity, % (90% CI)**	**Accuracy, % (90% CI)**
RF	1.00	100	100	1001	0.99 (0.95-1)	96 (89-100)	96 (88-100)	96 (91-100)
LDA	0.99	98	97	98	0.99 (0.97-1)	97 (90-100)	100 (92-100)	97 (92-100)
SVM	1.00	99	100	99	0.99 (0.96-1)	97 (90-100)	95 (89-100)	97 (92-100)
NBC	0.99	98	96	97	0.98 (0.95-1)	96 (85-100)	95 (85-100)	95 (88-99)
MLR	1.00	96	99	97	0.98 (0.88-1)	97 (89-100)	95 (82-100)	95 (88-100)

**Figure 3:**
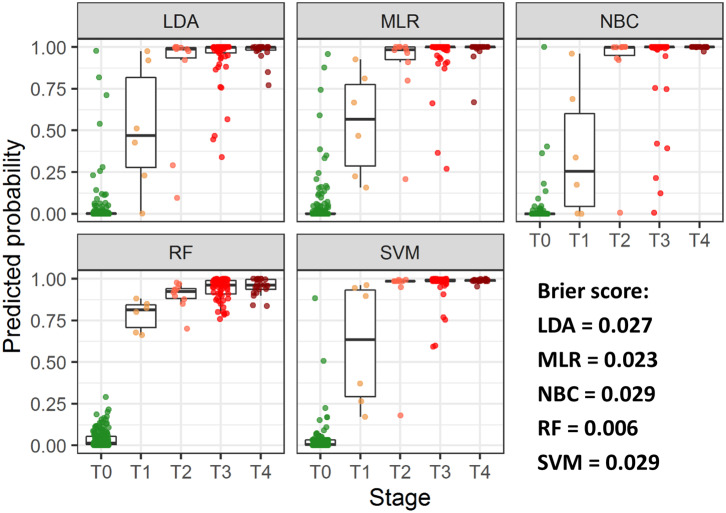
Predicted individual probabilities of having the disease stratified by CRC stage. Different stages are shown by color.

ROC analysis, performed separately on data, collected from patients with early and advanced disease stages, indicated higher performance of MLR, NBC and LDA classifiers for the latter group (Figure S2, Table S4). To further investigate diagnostic performance of the models for each cancer stage, individual probabilities of having the disease were calculated using the models, grouped by stage and visualized (Figure 3). All models correctly identified most of patients with T2-T4 stages, but patients with T1 were correctly classified only using RF model; this model also demonstrated the highest predictive accuracy (Brier score = 0.006).

**Figure 4:**
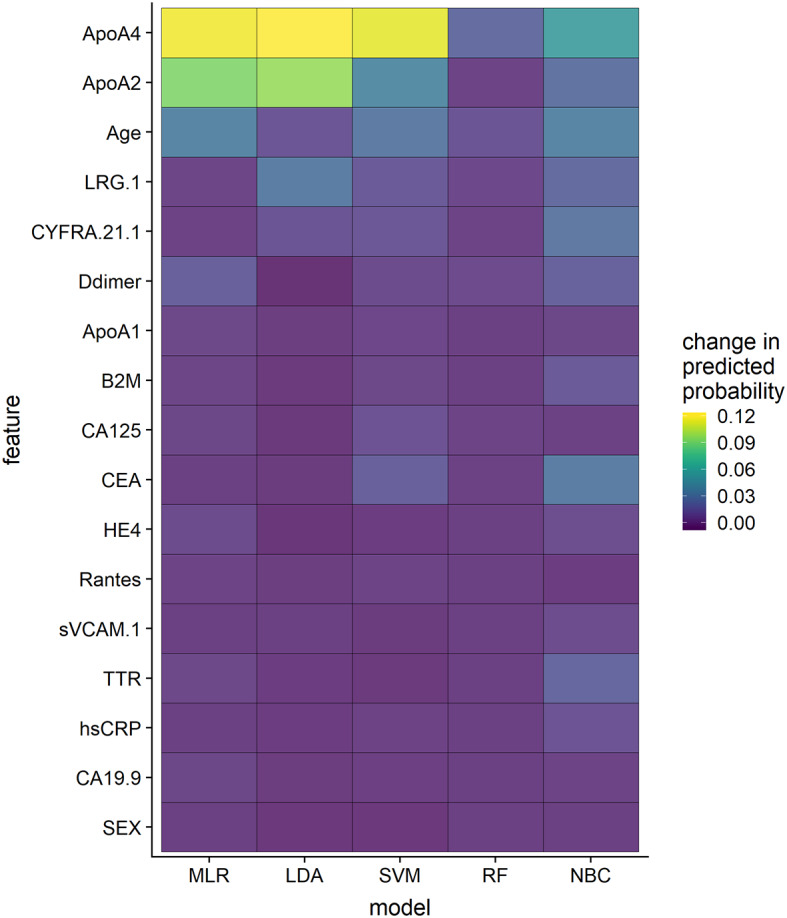
Feature importance measures for proposed classification models.

Sensitivity analysis revealed differences in feature importance across the developed models (Figure 4). Among tested classifiers RF classifier was less sensitive to feature permutations. Probabilities calculated using MLR, LDA, and SVM classifiers were sensitive to permutations in ApoA4 and ApoA2 levels; age was found to be an important patient characteristic for most of the tested algorithms.

### Testing Alternative Multivariate Classification Models

Our next question was to see whether a comparable diagnostic performance can be achieved by including information from lower number of biomarkers. To test this hypothesis, we selected SVM and LDA classifiers, and trained them using measurements of 1–5 biomarkers extracted from the whole dataset; influence of patient characteristics information inclusion into the models was additionally evaluated. In total, 6,340 models were tested, AUROC, sensitivity, and specificity was calculated.

**Figure 5:**
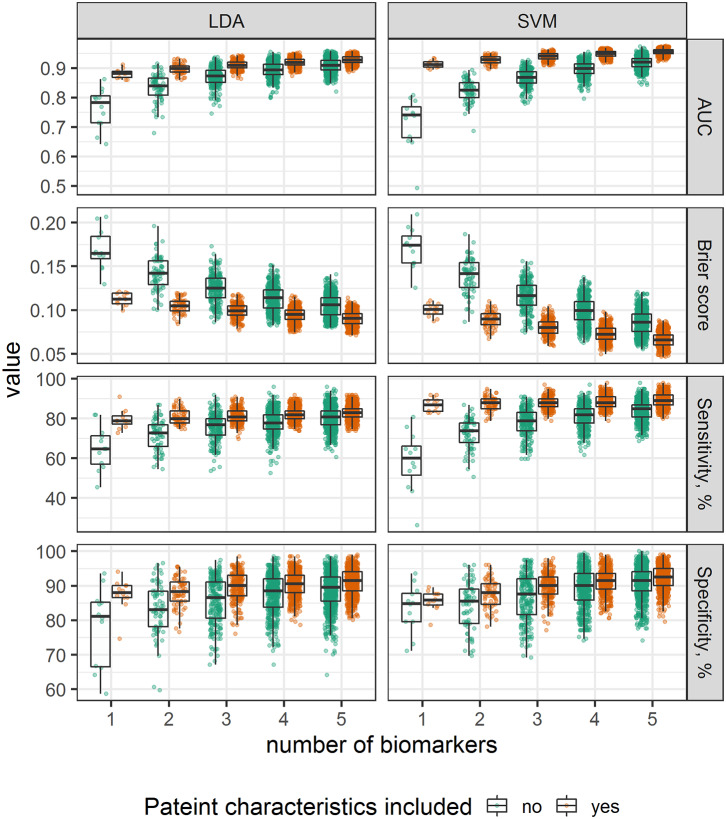
Comparison of alternative classification models, stratified by number of biomarkers and grouped by inclusion of age and gender.

Inclusion of information from higher number of biomarkers was followed by AUROC, sensitivity and specificity increase; taking into consideration the information about patient age and gender improved diagnostic performance of all combinations, mostly by increasing test sensitivity; this improvement is more pronounced in SVM vs. LDA algorithm, as a result, while accounting for patient characteristics, SVM performance was higher than LDA (Figure 5). While evaluating the discriminative ability, it was found that models, jointly considering information about both tumor antigens (e.g., CEA) and metabolic or inflammatory markers (e.g., ApoA2) demonstrated the highest diagnostic potential (Table 3).

**Table 3:** Diagnostic performance of 2-5-biomarker models for CRC diagnosis with highest AUROC values.

***N* of markers**	**Classification algorithm**	**Markers**	**AUROC**	**Specificity, %**	**Sensitivity, %**	**Accuracy, %**
2	SVM	CEA, hsCRP	0.96	88	90	88
2	LDA	CEA, ApoA2	0.96	96	81	91
3	LDA	CEA, B2M, ApoA2	0.95	95	82	91
3	SVM	hsCRP, CYFRA 21-1, ApoA2	0.97	93	89	91
4	LDA	CEA, B2M, Ddimer, ApoA2	0.96	97	81	92
4	SVM	CA 125, hsCRP, CYFRA 21-1, ApoA2	0.97	93	92	93
5	LDA	CEA, CA 125, B2M, Ddimer, ApoA2	0.96	93	86	90
5	SVM	CA 19-9, CA 125, B2M, ApoA1, ApoA2	0.98	91	93	91

As among 15 analytes, levels of ApoA2, ApoA4, Ddimer, HE4, and LRG 1 were found to be altered in patients with both early and advanced CRC stages (Figure 1), diagnostic performance of the combination of these 5 biomarkers was additionally evaluated and was shown to be comparable to that of the full 15-biomarker models (Table S5).

## Discussion

Multivariate approach represents a promising strategy to improve performance of diagnostic tools for cancer risk evaluation and several tests have been already approved by FDA, including OVA1® intended for ovarian cancer detection based on plasma measurements of 5 biomarkers (23), and multitarget stool DNA-based test Cologuard® for colorectal cancer screening (24). At the same time identification of new biomarkers in genome and proteome studies could further enhance the potential of cancer diagnostics (25, 26) whereas the increase of computational power followed by dissemination of machine learning techniques enabled a more efficient use of routinely collected patient data to improve different aspects of CRC screening. Hence, algorithms enabling identification of subjects with high CRC risk based on age, gender and full blood count information, can be applied to optimize screening programs (27–29), while deep learning methods could be used for computer-assisted colonoscopy image analysis (30). However, the development of multiple-biomarker tests still seems to be key to machine learning application in cancer diagnostics. In total, in a systematic review by Bhardwaj et al. 36 studies evaluated diagnostic performance of multiple-biomarker tests for CRC detection were identified (10). Variability in diagnostic performance of both single biomarkers and multiplex biomarker panels across the studies was reported, which was hypothesized as being a result of between-population differences as well as study design features (e.g., stage and histology of the tumors), thus, underlying the importance of developing or validating diagnostic platforms using the data obtained from intended to screen population. In the current study we reported the results of the cancer screening program “OncoPro,” aimed at improving early CRC detection in the Russian Federation.

Well-known biomarkers, associated with CRC diagnosis, such as CEA and CA 19-9 (31), demonstrated limited sensitivity in the present analysis and were not significantly increased in patients with early T1-T2 stages. This is in line with previous findings, which limits their usage in screening programs (32). Moreover, other proteins associated with CRC diagnosis such as CYFRA 21-1, HE 4, and LRG 1 were also tested and found to be altered in CRC patients, as previously reported (33–35). An interesting finding from the current study were the differences in PSA levels between healthy subjects and patients with CRC (1.13 ± 0.97 vs. 1.9 ± 1.61, *p*-value = 0.003), although the PSA level was only outside the reference range in two patients. One possible explanation could be the cross-reactivity of the PSA antibody with other serine proteases produced by colon cancer (36). Interestingly, in contrast to the results of the Hou, Luo, and Zhang meta-analysis (37), we found no AFP abnormalities in cancer subjects, which may suggest the need for screening tests adjusted to different populations. While the diagnostic potential of various antigens for CRC screening has been investigated, to our knowledge the current study is the first that demonstrates the alternations of metabolic markers ApoA1, ApoA2, and ApoA4 in CRC patients. Currently, ApoA1 is included into FDA-approved OVA1 test, used for ovarian cancer screening and was shown to be decreased in pancreatic cancer (38). These observations may point to antitumor ApoA1 activity (38), and support the link between metabolic disorders and cancer risk, previously hypothesized and investigated in the epidemiological Malmo Diet and Cancer Study (39).

The next step of our research was to evaluate the multivariate classification models, and in order to achieve this, we tested several classification algorithms, including information about different combinations of the aforementioned biomarkers, as well as patient characteristics. As expected, the diagnostic performance of multivariate models was higher compared to that of single-biomarkers and a number of considered biomarkers and patient characteristics was positively associated with the diagnostic accuracy of the tests. Classification models, exploiting information about all 15 biomarkers, age and gender of patients, demonstrated high performance (AUROC > 0.95) in line with previous studies, where similar biomarker panels enabled accurate identification of subjects with breast and lung cancer (40, 41). We hypothesized that such a good agreement between the model predictions and actual data could be consequence of overfitting, negatively affecting model predictive power, which is common for genomic and proteomic tests, exploiting information about thousands of predictors (42). A relatively small number of analytes was considered in the proposed models (15 biomarkers, age, and gender of patients) and cross-validation did not indicate this problem. Alternative explanation of good diagnostic performance of the models could be a large proportion of patients with advanced cancer stages, characterized by more pronounced alternations in biomarker levels. To evaluate this hypothesis, we investigated diagnostic performance of the models for early and advanced stages separately and compared posterior probabilities of the disease presence by stage. Higher probabilities were predicted for patients with advanced cancer stages using all classifiers, but only RF enabled accurate identification of patients with T1 stage. A possible explanation could be that this algorithm has more flexible structure compared to linear classifiers, such as MLR or LDA (43), howbeit, it should be stated, that performance of the algorithms may significantly depend on the tuning parameters (*e.g*. number of trees for RF or type of kernel function for SVM) and characteristics of a training dataset.

Whereas numerous multi-marker diagnostics tests with good performance have been developed already, they are not suitable for screening programs due to expensiveness. Cost-effective analysis did not demonstrate advantage of ~$500 Cologuard® test over current screening strategies (44). The estimated cost of the 15 biomarker-based analysis is ~$100, which is much cheaper compared to recently proposed multivariate diagnostic systems. To investigate possibility of further cost reduction, we evaluated models, considering smaller number of analytes, and identified several perspective candidates with good diagnostic performance.

As the current study was a pilot to evaluate the multiple-biomarker approach for CRC screening in the Russian Federation further research is still required to understand better the potential of the proposed classification models. This includes: (1) additional enrollment of patients with T1-T2 CRC stages, since the group size was relatively small in the current analysis; (2) inclusion of patients with benign tumors and colon diseases to evaluate the discriminative ability of the tests between CRC and other pathologies. Finally, prospective randomized clinical trials are required to demonstrate the clinical value of the proposed approach (42).

In conclusion, it could be stated that combinatorial biomarkers ensure more accurate discrimination between healthy subjects and CRC patients compared to univariate biomarkers and could be used as a decision-support tool for screening programs, however, further large-scale studies are necessary to confirm clinical utility of the developed diagnostic platform.

## Data Availability Statement

The datasets generated for this study are available on request to the corresponding author.

## Ethics Statement

The studies involving human participants were reviewed and approved by Local Ethics Committee of I.M. Sechenov First Moscow State Medical University. The patients/participants provided their written informed consent to participate in this study.

## Author Contributions

MS, PG, AS, EP, PT, AE, EG, and AR developed study concept and design. VV performed statistical data analysis and modeling and prepared a manuscript draft. All authors performed manuscript revision and made a substantial contribution to the research.

## Conflict of Interest

MS, PG, AS, EP, PT, AE, EG, and AR are currently applying for a patent relating to the models reported in the manuscript. VV is employed by M&S Decisions LLC and received research funding from AstraZeneca. The remaining authors declare that the research was conducted in the absence of any commercial or financial relationships that could be construed as a potential conflict of interest.
