# Supplementary material for: Diagnostic Value of Combinatorial Markers in Colorectal Carcinoma
Source: Front Oncol. 2020 May 22;10:832. doi: 10.3389/fonc.2020.00832 (PMC7258084; doi:10.3389/fonc.2020.00832)
Supplement: Supplementary file 1 [file Data_Sheet_1.docx]

**Table S1.** ANCOVA results: Age and gender impact on log-transformed biomarker levels

| Marker | pvalue, group | pvalue, gender | pvalue, age |
| --- | --- | --- | --- |
| LRG.1 | <0.001 | <0.001 | <0.001 |
| ApoA4 | <0.001 | 3.20E-01 | 3.10E-01 |
| ApoA2 | <0.001 | 1.40E-01 | <0.001 |
| B2M | <0.001 | 7.70E-01 | <0.001 |
| CYFRA.21.1 | <0.001 | 1.70E-01 | <0.001 |
| HE4 | <0.001 | 4.50E-02 | <0.001 |
| Ddimer | <0.001 | 1.10E-04 | <0.001 |
| TTR | <0.001 | 1.60E-07 | <0.001 |
| hsCRP | <0.001 | 2.40E-01 | 2.50E-02 |
| CEA | <0.001 | 1.20E-02 | 2.60E-02 |
| sVCAM.1 | <0.001 | 5.00E-01 | <0.001 |
| ApoA1 | <0.001 | <0.001 | 4.40E-01 |
| CA125 | <0.001 | 3.70E-03 | 8.80E-01 |
| CA19.9 | <0.001 | 4.50E-01 | 1.00E-01 |
| Rantes | 0.0024 | 1<0.001 | 1.60E-01 |
| ApoB | 0.17 | 3.30E-01 | 3.90E-01 |
| AFP | 0.25 | 3.90E-02 | 6.30E-01 |
| CA15.3 | 0.41 | 7.20E-01 | 4.10E-01 |
| VEGFR1 | 0.95 | 2.50E-01 | <0.001 |

**Table S2.** Diagnostic performance of biomarkers for CRC diagnosis at early (T1-T2) stages

| Biomarker | Units | Healthy subjects | CRC patients | p-value^a^ | AUROC | Specificity, % | Sensitivity, % | Accuracy, % |
| --- | --- | --- | --- | --- | --- | --- | --- | --- |
| AFP | U/ml | 2.84±1.96 | 2.87±2.07 | 1 | 0.52 | 91 | 25 | 86 |
| ApoA1 | g/l | 1.6±0.24 | 1.49±0.3 | 1 | 0.59 | 75 | 50 | 74 |
| ApoA2 | g/l | 0.3±0.04 | 0.24±0.05 | 0.022 | 0.76 | 99 | 44 | 95 |
| ApoA4 | mg/l | 69.22±16.88 | 35.83±15.74 | <0.001 | 0.92 | 88 | 81 | 88 |
| ApoB | g/l | 1.03±0.25 | 1.06±0.27 | 1 | 0.53 | 70 | 50 | 68 |
| B2M | ng/ml | 1477.29±293.83 | 1587.5±275.44 | 1 | 0.61 | 68 | 56 | 67 |
| CA125 | U/ml | 10.69±6.03 | 13.86±8.51 | 1 | 0.66 | 58 | 75 | 59 |
| CA15.3 | U/ml | 15.13±6.33 | 12.97±5.27 | 1 | 0.6 | 44 | 81 | 47 |
| CA19.9 | U/ml | 6.6±5.73 | 6.47±3.87 | 1 | 0.55 | 60 | 69 | 61 |
| CEA | ng/ml | 1.86±1.22 | 1.79±1.86 | 1 | 0.57 | 37 | 88 | 41 |
| CYFRA.21.1 | ng/ml | 1.37±0.57 | 1.53±0.72 | 1 | 0.55 | 91 | 25 | 86 |
| Ddimer | ng/ml | 119.75±103.78 | 652.03±1235.18 | 0.019 | 0.76 | 74 | 75 | 74 |
| HE4 | pM | 51.43±14.24 | 64.58±15.76 | 0.013 | 0.77 | 68 | 88 | 70 |
| hsCRP | mg/l | 1.77±3.51 | 4.21±6.65 | 1 | 0.63 | 85 | 38 | 81 |
| LRG.1 | ng/ml | 58847.79±25516.45 | 93607.12±39452.24 | 0.0069 | 0.78 | 77 | 69 | 76 |
| Rantes | pg/ml | 57217.76±23360.49 | 66753.31±38273.26 | 1 | 0.55 | 97 | 25 | 92 |
| sVCAM.1 | ng/ml | 658.37±131.52 | 712.44±152.38 | 1 | 0.61 | 83 | 44 | 80 |
| t.PSA | ng/ml | 1.13±0.97 | 2.08±1.34 | 1 | 0.72 | 83 | 71 | 82 |
| TTR | mg/dl | 25.64±4.88 | 20.88±6.66 | 0.36 | 0.7 | 93 | 38 | 89 |
| VEGFR1 | pg/ml | 122.76±24.08 | 140.12±48.47 | 1 | 0.57 | 86 | 31 | 82 |

**Table S3.** Diagnostic performance of biomarkers for CRC diagnosis at advanced (T3-T4) stages

| Biomarker | Units | Healthy subjects | CRC patients | p-value^a^ | AUROC | Specificity, % | Sensitivity, % | Accuracy, % |
| --- | --- | --- | --- | --- | --- | --- | --- | --- |
| AFP | U/ml | 2.84±1.96 | 2.71±2.16 | 1 | 0.55 | 47 | 67 | 53 |
| ApoA1 | g/l | 1.6±0.24 | 1.39±0.23 | <0.001 | 0.74 | 66 | 73 | 68 |
| ApoA2 | g/l | 0.3±0.04 | 0.22±0.04 | <0.001 | 0.89 | 94 | 69 | 86 |
| ApoA4 | mg/l | 69.22±16.88 | 40.4±14.69 | <0.001 | 0.9 | 74 | 93 | 80 |
| ApoB | g/l | 1.03±0.25 | 1.06±0.24 | 1 | 0.56 | 64 | 51 | 60 |
| B2M | ng/ml | 1477.29±293.83 | 2152.09±692.64 | <0.001 | 0.88 | 75 | 86 | 78 |
| CA125 | U/ml | 10.69±6.03 | 17.38±23.56 | 0.014 | 0.63 | 67 | 54 | 64 |
| CA15.3 | U/ml | 15.13±6.33 | 15.49±8.28 | 1 | 0.51 | 76 | 32 | 63 |
| CA19.9 | U/ml | 6.6±5.73 | 21.2±40.14 | <0.001 | 0.68 | 86 | 52 | 76 |
| CEA | ng/ml | 1.86±1.22 | 56.5±334.8 | <0.001 | 0.81 | 92 | 65 | 84 |
| CYFRA.21.1 | ng/ml | 1.37±0.57 | 4.52±6.48 | <0.001 | 0.87 | 79 | 79 | 79 |
| Ddimer | ng/ml | 119.75±103.78 | 398.19±627.37 | <0.001 | 0.81 | 64 | 84 | 70 |
| HE4 | pM | 51.43±14.24 | 79.53±36.17 | <0.001 | 0.81 | 67 | 81 | 71 |
| hsCRP | mg/l | 1.77±3.51 | 12.68±18.05 | <0.001 | 0.82 | 85 | 66 | 79 |
| LRG.1 | ng/ml | 58847.79±25516.45 | 126610.21±48196.69 | <0.001 | 0.91 | 82 | 88 | 84 |
| Rantes | pg/ml | 57217.76±23360.49 | 69564.86±26728.4 | 0.0022 | 0.65 | 68 | 61 | 66 |
| sVCAM.1 | ng/ml | 658.37±131.52 | 876±308.3 | <0.001 | 0.74 | 85 | 54 | 76 |
| t.PSA | ng/ml | 1.13±0.97 | 1.87±1.66 | 0.018 | 0.69 | 80 | 62 | 75 |
| TTR | mg/dl | 25.64±4.88 | 18.98±6.54 | <0.001 | 0.78 | 85 | 59 | 77 |
| VEGFR1 | pg/ml | 122.76±24.08 | 124.01±42.44 | 1 | 0.53 | 87 | 29 | 70 |

**Table S4.** Diagnostic performance of performance of 15-biomarker models at early and advanced CRC stages

| Early stages | | | | |
| --- | --- | --- | --- | --- |
| Method | AUROC | Specificity, % | Sensitivity, % | Accuracy, % |
| RF | 1.00 | 100 | 100 | 100 |
| LDA | 0.97 | 95 | 94 | 94 |
| SVM | 1.00 | 99 | 100 | 99 |
| NBC | 0.97 | 82 | 100 | 83 |
| MLR | 0.99 | 94 | 100 | 94 |
| Advanced stages | | | | |
| Method | AUROC | Specificity | Sensitivity | Accuracy |
| RF | 1.00 | 100 | 100 | 100 |
| LDA | 1.00 | 98 | 100 | 99 |
| SVM | 1.00 | 100 | 100 | 100 |
| NBC | 1.00 | 98 | 99 | 98 |
| MLR | 1.00 | 97 | 100 | 98 |

**Table S5.** Diagnostic performance of 5-biomarker (ApoA2, ApoA4, Ddimer, HE4 and LRG 1) models at all, early and advanced CRC stages

| All stages | | | | |
| --- | --- | --- | --- | --- |
| Method | AUROC | specificity, % | sensitivity, % | accuracy, % |
| RF | 1.00 | 100 | 100 | 100 |
| LDA | 0.99 | 95 | 94 | 95 |
| SVM | 1.00 | 99 | 95 | 97 |
| NBC | 1.00 | 97 | 100 | 98 |
| MLR | 1.00 | 96 | 99 | 97 |
| Early stages | | | | |
| Method | AUROC | specificity, % | sensitivity, % | accuracy, % |
| RF | 1.00 | 100 | 100 | 100 |
| LDA | 0.95 | 92 | 94 | 92 |
| SVM | 0.99 | 91 | 100 | 91 |
| NBC | 0.99 | 97 | 100 | 97 |
| MLR | 0.97 | 86 | 100 | 87 |
| Late stages | | | | |
| Method | AUROC | specificity, % | sensitivity, % | accuracy, % |
| RF | 1.00 | 100 | 100 | 100 |
| LDA | 0.99 | 97 | 98 | 97 |
| SVM | 1.00 | 99 | 98 | 98 |
| NBC | 1.00 | 99 | 99 | 99 |
| MLR | 0.99 | 95 | 98 | 96 |


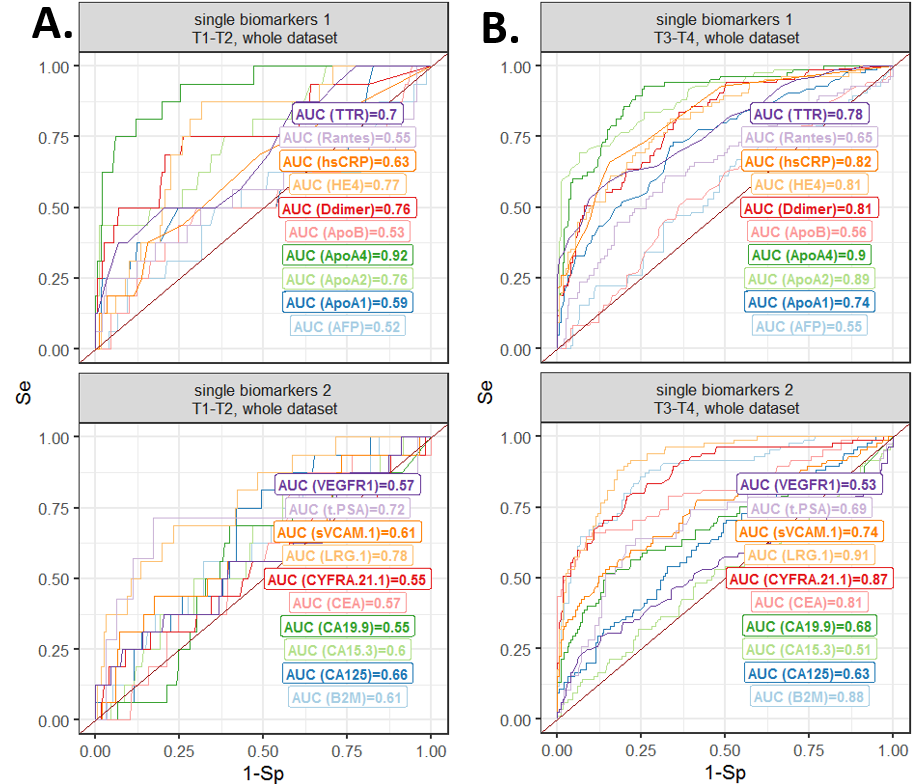


**Figure S1.** ROC curves for the single-biomarker based tests at **(A)** early (T1-T2) and **(B)** advanced (T3-T4) CRC stages. Different biomarkers are shown by color. Numbers denote AUROC values


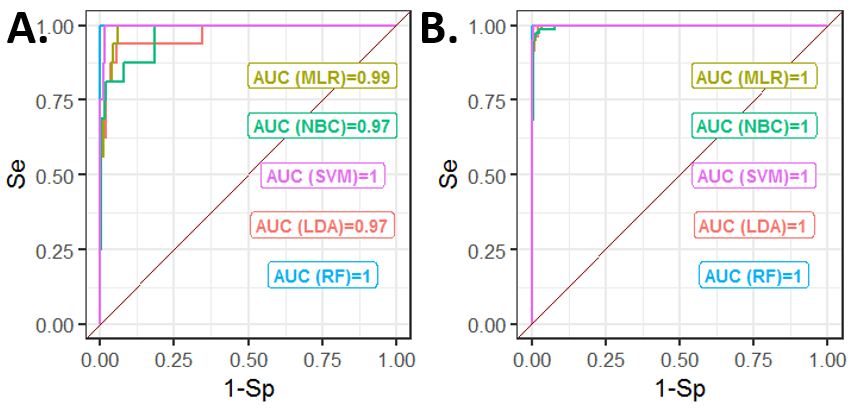


**Figure S2.** ROC curves for the multivariate classification models at **(A)** early (T1-T2) and **(B)** advanced (T3-T4) CRC stages. Different biomarkers are shown by color. Numbers denote AUROC values.
